# Oxidative Stress Promotes Axonal Atrophy through Alterations in Microtubules and EB1 Function

**DOI:** 10.14336/AD.2024.0839

**Published:** 2025-12-03

**Authors:** Samuel Shields, Emilia Gregory, Oliver Wilkes, IIlana Gozes, Natalia Sanchez-Soriano

**Affiliations:** ^1^Institute of Systems, Molecular and Integrative Biology, University of Liverpool, UK.; ^2^The Elton Laboratory for Molecular Neuroendocrinology, Department of Human Molecular Genetics and Biochemistry, Faculty of Medical & Health Sciences, Sagol School of Neuroscience and Adams Super Center for Brain Studies, Tel Aviv University, Tel Aviv 6997801, Israel.

**Keywords:** Oxidative stress, reactive oxygen species, aging, microtubules, neuron, axons, swellings

## Abstract

Axons are crucial for transmitting neurochemical signals. As organisms age, the ability of neurons to maintain their axons declines; hence, aged axons are more susceptible to damage or dysfunction. Understanding how aging causes axonal vulnerability is crucial for developing strategies to enhance overall resilience of neurons and prevent neuronal deterioration during aging and in age-related neurodegenerative diseases. Increasing levels of reactive oxygen species (ROS) causes oxidative stress — a hallmark of aging and age-related diseases. Despite this association, a causal relationship between oxidative stress and neuronal aging remains unclear, particularly in how subcellular physiology may be affected by ROS. By using *Drosophila*-derived primary neuronal cultures and a recently developed *in vivo* neuronal model of aging, which involves the visualisation of *Drosophila* medulla neurons, we investigated the interplay between oxidative stress, neuronal aging and the microtubule cytoskeleton. Our results showed that oxidative stress is a key driver of axonal and synaptic decay, as shown by an enhanced appearance of axonal swellings, microtubule alterations (in both axons and synapses) and morphological transformation of axonal terminals during aging. We demonstrated that increasing the levels of ROS sensitises microtubule plus end-binding protein 1 (EB1), leading to microtubule defects that effect neuronal integrity. Furthermore, manipulating EB1 proved to be a valuable therapeutic strategy to prevent aging hallmarks enhanced in conditions of elevated ROS. In summary, we demonstrate a mechanistic pathway linking cellular oxidative stress with changes in the microtubule cytoskeleton leading to axonal deterioration during aging and provide evidence of the therapeutic potential of enhancing microtubule plus-end physiology to improve the resilience of axons.

## INTRODUCTION

Aging is associated with a decline in cognition, motor and sensory function, impacting activities of daily living and quality of life [1]. The determinants and cellular processes of aging in the brain are not well understood, highlighting the need for in-depth study of this process.

Reactive oxygen species (ROS) are molecules produced as a by-product of respiration with the capacity to be deleterious in high concentrations. An array of molecular (e.g. glutathione [GSH], vitamin E) and enzymatic (e.g. superoxide dismutases [SODs], catalase) antioxidants act to control ROS levels throughout cells. Increasing levels of ROS (oxidative stress) classically correlate with age in humans and animal models [2-6]. Oxidative stress is also implicated in the pathology of various neurodegenerative diseases, including Alzheimer’s disease (AD), Parkinson’s disease (PD) and amyotrophic lateral sclerosis (ALS) [7]. Recent studies demonstrated that ROS are akin to second messengers, capable of regulating many downstream signalling functions. During aging, oxidative stress may perturb redox signalling and homeostasis and, therefore, negatively impact cellular function; also known as the redox theory of aging [5, 8]. While the redox theory of brain aging has gained support in recent years, the specific cellular and molecular mechanisms whereby oxidative stress affects aging neurons are not understood.

Microtubules (MTs) are filamentous polymers, comprising non-covalent longitudinal arrangements of α- and β-tubulin heterodimers, and are a key constituent of the cytoskeleton [9]. MT integrity is required for the proper functioning of the nervous system. In axons, MTs are organised in densely packed parallel bundles and act as key proponents of axonal structural resistance and morphology by influencing axon calibre [10-12]. MTs also facilitate the transport of essential material, including organelles, RNAs, proteins and synaptic vesicles in neurons [13, 14]. Dysregulation of the MT cytoskeleton has been reported in pyramidal neurons found in aged human brains and in axons of aged Resus monkey brains [15, 16]. Our group has shown that similar alterations in MT density and organisation are found in medulla neurons situated in the *Drosophila* brain, and that these changes are true hallmarks of aging in neurons [12]. Given that perturbed MT function is also linked to a plethora of age-related neurodegenerative diseases, MT deterioration could be a key event driving neuronal vulnerability in brain aging and neurodegenerative conditions. Yet, what causes such alterations on MTs in neurons and their axons during aging lacks comprehensive exploration.

*In vitro* studies of myocytes show that increased ROS can induce alterations in the properties of MTs [17, 18]. This observation led us to hypothesise that neuronal deterioration in aging could be propelled by oxidative stress, adversely affecting the function of MTs. To test this, we used a previously described model used to study the cell biology of neuronal aging in the *Drosophila* optic lobe. We found that chemical inducers of oxidative stress, or genetic reduction of enzymatic (SOD1 and SOD2) or non-enzymatic antioxidants (GSH), exacerbates aging hallmarks, including the appearance of axonal swellings and bulbous synaptic terminals. We also showed that enhancing the antioxidant response in neurons by manipulating kelch-like ECH-associated protein 1 (Keap1) levels, a redox-sensitive direct inhibitor of nuclear factor erythroid 2-related factor 2 (NRF2), attenuated aging hallmarks. Importantly, we discovered that oxidative stress induces MT dysfunction *in vitro* and *vivo*, suggesting a key role of oxidative stress in the process of aging. Furthermore, the observed changes in MT organisation, caused by high levels of ROS, were caused by impaired end-binding protein 1 (EB1) activity, and enhancing EB1 levels was successful in preventing ROS-induced, age-related neuronal deterioration. This new understanding of the intricate interplay between aging, oxidative stress and the MT cytoskeleton may offer novel therapeutic strategies to safeguard neuronal integrity as individuals age.

## MATERIALS AND METHODS

### Fly stocks and husbandry

Flies were maintained on standard fly food containing cornflour, glucose, yeast and agar. For experiments involving aging, flies were maintained at 25°C during development and transferred to 29°C post-eclosion to expedite the aging process. Flies were maintained at low densities (maximum of 20 flies per vial) and were transferred to new vials with fresh food every 3 days. Both males and females were used for aging experiments. Flies within each vial were of the same sex and all analytical comparisons were age and sex matched accordingly.

Gal4-driver lines used were *elav-Gal4* (3^rd^ chromosomal, expressing pan-neuronally at all stages [19]) and *GMR31F10-Gal4* (Bloomington stock 49685; expressing in T1 medulla neurons; [20]). Mutant alleles used were: *SOD1^n1^* [21] (Bloomington #24492), *SOD1^n64^* [21] (Bloomington #7451), *SOD2^Δ02^* (Bloomington #27643) and *EB1^04524^* [22]. Lines used for overexpression experiments were *UAS-GFP-α-tubulin84B* [23], *UAS-myr::tdTomato* (Bloomington #32222), *UAS-catalase* [24], *UAS-EB1::GFP* and *UAS-EB1::mCherry* [25]. The lines used for knockdown experiments were *SOD1 RNAi* (Bloomington #36804), *Gss1 and Gss2 RNAi* (Bloomington #55150) and *Keap1 RNAi* (Bloomington #57801).

### Adult drug feeding

Paraquat (PQ;1,1′-dimethyl-4,4′-bipyridinium dichloride; Merck) and diethyl maleate (DEM; diethyl (*Z*)-but-2-enedioate; Alfa Aesar) feeding protocols were adapted from [26]. Flies were maintained for approximately 14 days (middle age) prior to drug feeding. Flies were then transferred to empty food vials containing layered 188 mm Whatman™ filter paper soaked with 500 μL drug/vehicle diluted in 2.5% sucrose (Fisher)/Dulbecco’s phosphate-buffered saline (Sigma, RNBF2227). Flies were exposed to the test drug for 24 hours before transferring back to standard fly food for a further 24 hours (without the drug). This 24-hour cycle was repeated until flies had received a total of four 24-hour doses (over an 8-day period). Flies were then prepared for brain dissections and confocal imaging (*described below*).

### Live adult brain dissections and microscopy

*Drosophila* brains were dissected in Dulbecco's PBS after initial anaesthetisation on ice for 1.5 minutes; this method of anaesthetisation has been shown to not affect MT physiology in T1 cells [12]. Dissected brains were mounted in Dulbecco's PBS on MatTek glass-bottom dishes (P35G1.5-14C) with a spacer and coverslip. Brains were immediately imaged using a 3i Marianas Spinning Disk with a 63× 1.4 NA Zeiss Plan Apochromat lens and FLASH4 sCMOS (Hamamatsu) camera (incubated at 26ºC); z-stacks of whole brains were taken using a slice interval of 0.3 µm. All imaging was conducted at the Centre for Cell Imaging at the University of Liverpool. For each experimental repeat, the ratio of male and female brains was kept consistent, and data were normalised to respective controls.

### Brain image analyses

Using FIJI/ImageJ, several maximum projection images were generated from captured z-stacks to facilitate accurate visualisation and analysis of layered T1 axons and axon terminals. For consistency, analyses of axonal membranes (e.g. membrane swellings) and MT-related phenotypes were restricted to the same regions of axon bundles. The number of axonal swellings and regions of disorganised MTs were recorded and presented per nerve bundle.

For analyses of axon terminals, maximum-projection images of the terminal compartments were generated from z-stacks. A 70 µm × 70 µm area was outlined in the centre of each medulla for analysis, given most axon terminals were easily identifiable in this region (approximately 30-50 synaptic terminals per medulla were captured). The proportion of axon terminal swellings and terminals with fragmented/broken MTs per medulla and the mean number of synaptic MTs per terminal were quantified. For qualitative measurements (i.e. swollen and fragmented phenotypes), data were presented as a percentage of all terminals evaluated per medulla. For axonal diameter, maximum-projection images were generated and oriented to view axons in parallel alignment to facilitate consistency in the analysis. Images containing only the membrane channel were processed using the ‘tubeness’ plugin available in FIJI/ImageJ. To eliminate biases in measuring the diameter of axonal membranes, a 10 µm × 10 µm grid was overlayed and measurements were obtained using the Fiji/ImageJ line tool at points where T1 neurons overlapped with the overlaid grid. The resulting values were used to calculate mean diameter per axon.

### Primary neuronal cell culture and drug treatments

*Drosophila* primary neuronal cultures were carried out using previously described methods [27, 28]. Drug treatments were carried out at 0-3 DIV, as stated throughout, according to the experimental approach. Compounds used were: 100 µM PQ (vehicle: H_2_O), 100 µM DEM (vehicle: ethanol), 100 µM Trolox ([±]-6-Hydroxy-2,5,7,8-tetramethylchromane-2-carboxylic acid; Merck), 100 µM nocodazole and 1 nM NAP (Illana Gozes, Tel Aviv University, Israel).

### Immunocytochemistry of cultured neurons

Unless otherwise stated, cultured neurons were fixed in 4% paraformaldehyde (PFA) in 0.05 M phosphate buffer (pH 7.0-7.2) for 30 minutes at room temperature. For analyses of EB1 comets, primary *Drosophila* neurons were fixed in ice-cold (stored at -80ºC) +TIP-fixative solution (90% methanol, 3% formaldehyde, 5 mM sodium carbonate, pH 9; [29]) for 10 minutes. Antibody staining and washes were performed in PBS supplemented with 0.3% Triton X-100.

Primary antibodies used were: Cy3-751 conjugated anti-HRP (goat, 1:100; Jackson ImmunoResearch), anti-α-tubulin (mouse, 1:1000; T9026, Merck), anti-DmEB1 (rabbit, 1:2000, gift from H. Ohkura). Secondary antibodies used were FITC- and Cy3-conjugated secondary antibodies (goat, 1:100; Jackson Immuno-Research, 123-095-021-JIR and 715-165-151-JIR). Cells were embedded in Vectashield (H-1000, VectorLabs).

### Microscopy of cultured neurons and image analyses

Fixed *Drosophila* cells were captured using a Nikon eclipse 90i with a Retiga 3000 camera (QImaging) or a 3i Marianas Spinning Disk using a 63× 1.4 NA Zeiss Plan Apochromat lens and FLASH4 sCMOS (Hamamatsu) camera at the Centre for Cell Imaging at the University of Liverpool. To measure the degree of MT disorganisation or curling in axons, a ‘MT-disorganisation index (MDI)’ was used. The total area of MT disorganisation per axon (measured using the FIJI/ImageJ freehand selection tool) was divided by the respective axon length (measured using the segmented line tool) [27, 28]. EB1 comet lengths were measured using the FIJI/ImageJ line tool and data were presented as mean EB1 comet length per cell.

### Statistical analyses

Statistical analyses were performed in GraphPad Prism 9; statistical tests used are stated in figure legends and exact p-values are indicated in graphs. Sample sizes and other statistical values are indicated in figure legends.

## RESULTS

### Reductions in SOD activity or GSH levels induce microtubule defects and exacerbate axonal and synaptic aging hallmarks

To evaluate the impact of ROS on MTs and neuronal aging *in vivo*, we used a novel *Drosophila* model, previously established by our group [12]. This model facillitates the study of the cell biology of aging in T1 neurons, which reside in the medulla of the *Drosophila* optic lobe, with cell bodies localised at the outer medulla cortex. T1 axons, which terminate and synapse within the M2 layer, are easily resolved [12]. Alterations in axonal (swellings, reduced diameter) and synaptic morphology (swellings and fragmentation), and in MTs (foci with unbundled disorganised MTs and reductions in the number of MTs extending into synaptic terminals), altogether referred to as hallmarks of aging in this model, develop with age and begin to appear in T1 neurons within a few weeks of *Drosophila* life [12].

**Figure 1.**
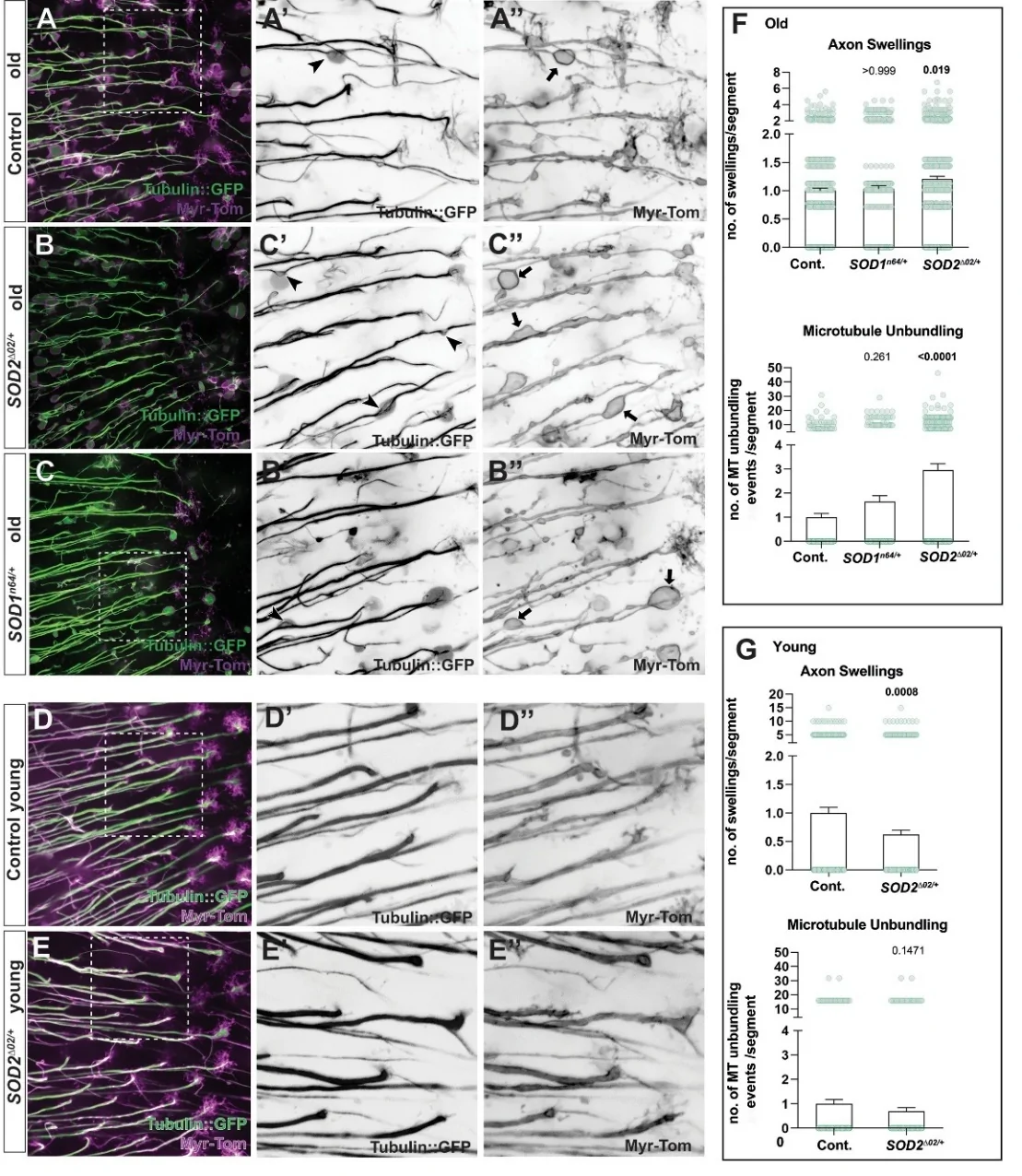
**Mutant SOD2 exacerbates neuronal aging hallmarks of axonal swellings and microtubule disorganisation in brains from aged *Drosophila***. (A-E’’) Medulla regions of adult brains aged 31-35 days (A-C) or 5-7 days (D-E) post eclosure, which show T1 axons labelled with GFP-tagged α-tubulin (tubulin::GFP) and the plasma membrane marker myristoylated-Tomato (myr-Tom). Magnified inverted-grayscale images of areas outlined by dashed white boxes, are shown for tubulin::GFP (A’, B’, C’, D’ and E’) and Myr-Tom (A’’, B’’, C’’, D’’ and E’’). Aged and young neurons in the absence (A and D) or presence of *SOD1^n64/+^* (C) or *SOD2^Δ02/+^* (B and E) mutant backgrounds are compared. *SOD2^Δ02/+^* enhance phenotypes in aging neurons, comprising axon swellings (black arrows) and MT unbundling (arrow heads), but not in young neurons. (F and G) Quantitative analyses of the frequency of axonal swellings and microtubule unbundling; bars represent normalised mean ± SEM; data points are shown in green; p values are shown above each bar, as assessed by Kruskal-Wallis one-way tests. Data were collated across four individual repeats, from a minimum of 355 axonal segments from 32 control medullas, 26 *SOD1^n64/+^* medullas and 33 *SOD2^Δ02/+^* medullas for old specimens. For young specimens, data were collated across three individual repeats, from a minimum of 555 axonal segments from 43 control medullas and 50 *SOD2^Δ02/+^* medullas. Scale bars = 10 μm.

**Figure 2.**
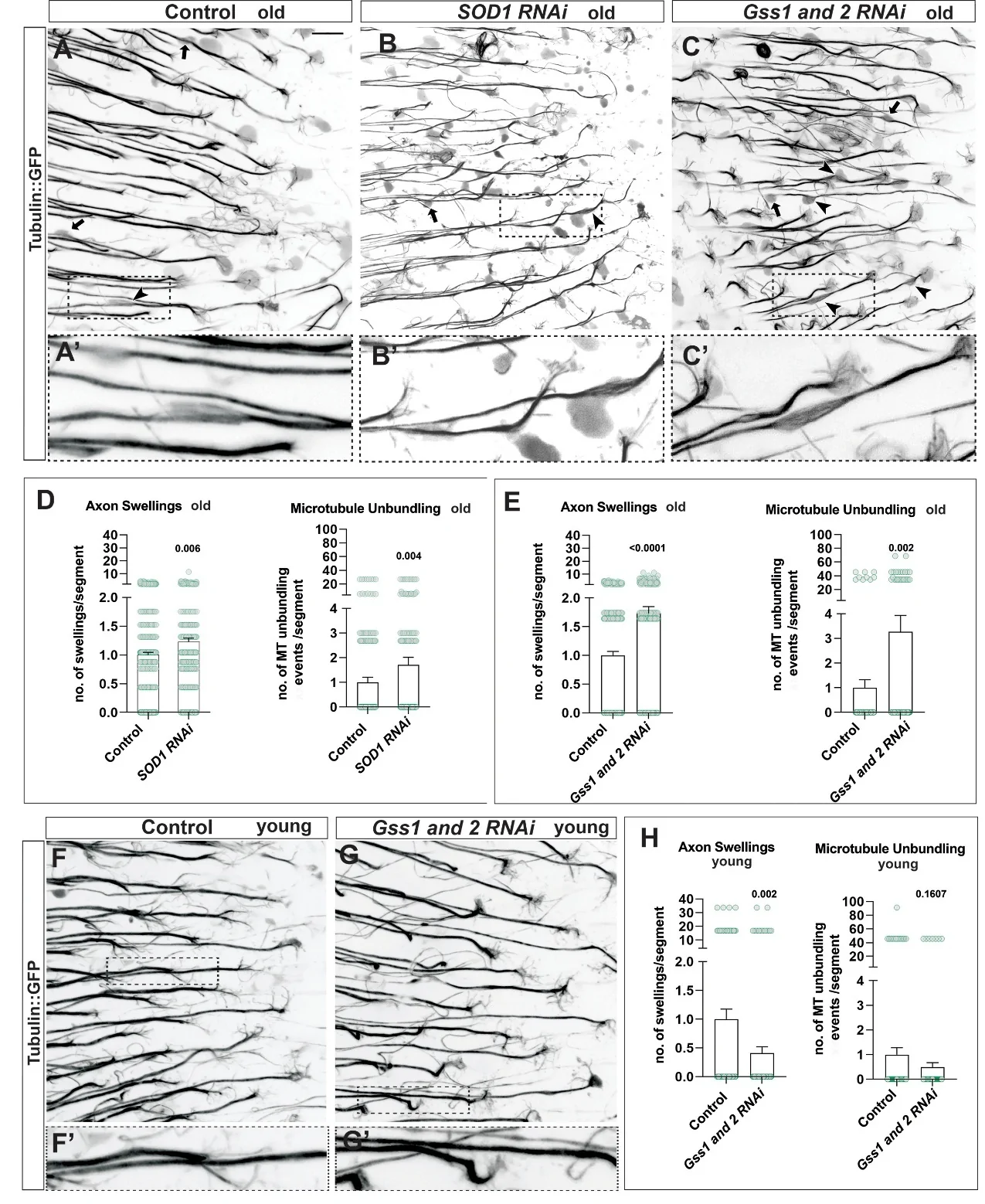
**Knocking down Gss1 and 2 and SOD1, enhances age-related axonal swellings and microtubule unbundling**. (A-C’ and F-G’) Medulla regions of adult brains aged 30-33 days or 5-7 days (F-G’) post eclosure, which show T1 axons labelled with GFP-tagged α-tubulin (tubulin::GFP) presented as inverted greyscale images. Aged neurons in the absence (A) or presence of *SOD1* knockdown *SOD1 RNAi*; (B) or *Gss1 and 2* knockdown (*Gss1 and 2 RNAi*; C) are compared. *SOD1* and *Gss1* and *2* knockdown, enhances phenotypes in aging neurons, such as axon swellings (arrows) and MT unbundling (arrow heads), but not in young neurons (F-G); dashed boxed areas are magnified and shown in A’, B’, C’, F’ and G’. (D, E and H) Quantitative analyses of the frequency of axonal swellings and microtubule unbundling for *SOD1* (D) and for *Gss1 and 2* (E and H) knockdown; bars represent normalised mean ± SEM; data points are shown in green; p values are shown above each bar, as assessed by Mann-Whitney rank sum tests. For old and young specimens, data were generated from across three individual repeats; from a minimum of 304 axonal segments from 23 control and 18 *SOD1 RNAi* medullas in D and 19 control and 19 *Gss1 and 2 RNAi* medullas in E, and from a minimum of 630 axonal segments from 46 control and 48 *Gss1 and 2 RNAi* medullas in H. Scale bars = 10 μm.

SODs are metalloenzymes that catalyse the conversion of superoxide radicals to hydrogen peroxide, which is then further reduced to water by peroxidases. SOD1 is predominantly cytosolic, whereas SOD2 is localised to mitochondria. We utilised two amorphic alleles of SODs, *SOD1^n64^* and *SOD2^Δ02^* [21, 30] to mimic oxidative stress. Since both alleles cause adult lethality in homozygosis, their effects in the T1 aging model were studied in heterozygosis. In this model, MTs were labelled with *UAS-α-Tubulin84B::GFP* and cell membranes with *UAS-myristoylated-tomato*, under the control of the T1-specific Gal4 driver, *GMR31F10-Gal4* [12].

We found that *SOD2^Δ02^*^/+^ flies aged 30-34 days displayed a significant, 3-fold increase in the frequency of unbundled/disorganised MTs within axons as well as a significant increase in the number of axonal swellings compared with age-matched controls (Fig. 1 A, B and F); however, there was no significant increase in the frequency of swellings or unbundled/disorganised MTs in young flies (4-7 days) (Fig. 1 D, E and G).

**Figure 3.**
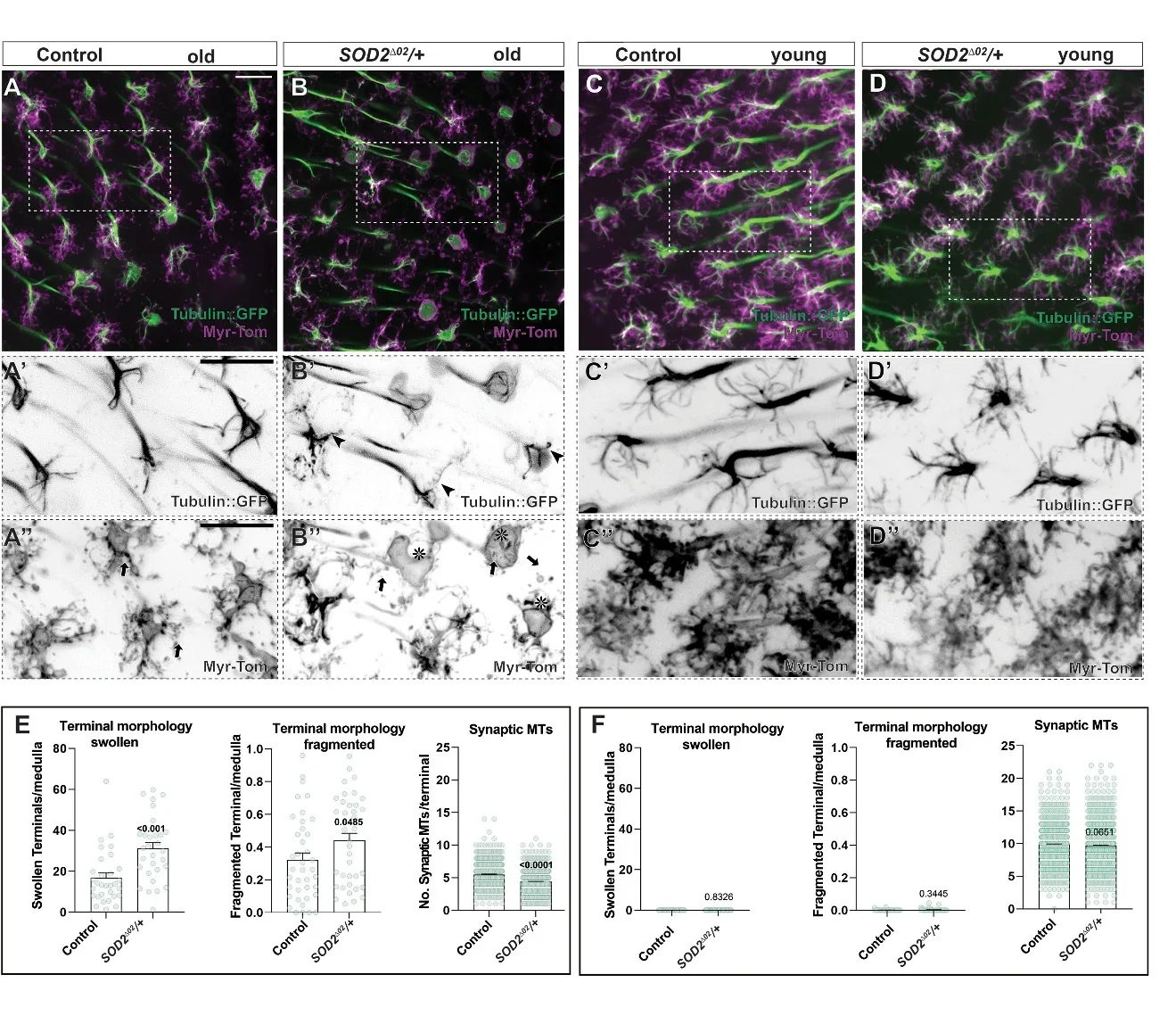
**Mutant SOD2 induces a decrease in synaptic microtubules and worsens the deterioration of T1 synaptic terminals in brains of aged *Drosophila***. (A-D’’) Axon terminals of T1-cell projections in the medulla of aged (31-35 days, A and B) or young (5-7 days, C and D) *Drosophila* post eclosure, labelled with GFP-tagged α-tubulin (tubulin::GFP) and the plasma membrane marker myristoylated-Tomato (myr-Tom). Magnified inverted-greyscale images of areas outlined by dashed boxes in A-D are, respectively, shown for tubulin::GFP (A’-D’) and Myr-Tom (A’’-D’’). Aging phenotypes at the synaptic terminals, including an increase in swollen (asterisks) and broken terminals (arrows), and a decrease in synaptic MTs (arrowheads), are enhanced in the presence of *SOD2^Δ02/+^* mutant backgrounds in old specimens but not in young ones. (E and F) Quantitative analyses of the frequency of swollen and fragmented terminals and the number of synaptic microtubules in control and *SOD2^Δ02/+^* mutant backgrounds; bars represent normalised mean ± SEM; data points are shown in green; p values are shown above each bar, as assessed by Mann-Whitney rank sum tests. For old specimens, data were generated from four independent repeats, from around 700 synapses from 31 control and 31 *SOD2^Δ02/+^* medullas. Data for young specimens were generated from 3 independent repeats, from around 900 synapses from 41 control and 53 *SOD2^Δ02/+^* medullas. Scale bars = 10 μm.

*SOD1^n64/+^* failed to significantly enhance axon swellings or MT unbundling in aged flies, despite increasing trends (Fig. 1 A, C and F). Since the expression of one wildtype copy of *SOD1* may provide sufficient function, we tested whether knockdown of *SOD1*, using a validated *SOD1 RNAi* strain (*SOD1^GL01016^* [31]), led to different outcomes; *SOD1* knockdown significantly increased the frequency of MT unbundling and axonal swellings in aged specimens (Fig. 2, A, B and D).

Age-related changes in T1 morphology have been observed at synaptic terminals, including the development of swollen or bulbous membranes, that can appear fragmented and the reductions in the number of invading MTs at synapse [12]. Given that *SOD2* had a stronger impact on axonal aging, we assessed whether similar effects were observed at the level of synapses in *SOD2^Δ02/+^* flies. Supportive of our axonal findings, *SOD2^Δ02/+^* flies had significantly higher proportions of swollen and fragmented membranes, and significantly fewer MTs visible in synapses, compared with aged-matched controls (Fig. 3, A, B and E); such alterations at synaptic terminals were not observed in young (4-7 days) specimens (Fig. 3, C, D and F).

Another layer of the antioxidant defence in cells is provided by small molecule, or non-enzymatic, antioxidants, which function as ROS scavengers or cofactors for ROS detoxification and metabolism. An integral redox couple and antioxidant in cells is GSH/GSSH. GSH synthetase (Gss) is an essential enzyme involved in *de novo* synthesis of GSH. We hypothesised that reducing GSH levels, via Gss1 and Gss2 knockdown, may impact physiological aging observed in the T1 model. Similar to the results observed with SOD2/SOD1, knockdown of *Gss1* and *Gss2* significantly exacerbated the frequency of MT unbundling and axonal swellings in old flies, and not in young flies when compared with age-matched controls, respectively (Fig. 2A, C, E and F-H). These data demonstrate that diminishing antioxidant activity can alter basic MT properties in axons and exacerbate age-related changes in axon and synaptic morphology by cell autonomous mechanisms.

### Enhancing antioxidant responses by knockdown of *Keap1* protects against MT deterioration and axonal and synaptic decay during aging

Next, we investigated whether enhancing the antioxidant defence system could prevent negative consequences of aging on neuronal MTs and axon and synaptic morphological alterations. To gain this insight, we used two approaches: overexpression of a single enzymatic antioxidant, catalase, which is important for the elimination of H_2_O_2_ in cells [32, 33]; or upregulation of a network of antioxidant and redox-signalling pathways by knocking down *Keap1*, a repressor of NRF2 [34].

Flies overexpressing catalase had significantly more axonal swellings than aged-matched controls (31-34 days), and there was no change in the frequency of MT unbundling (Supplementary Fig. 1). This may suggest that some functional ROS are needed and, therefore, a fine balance in the levels of basal ROS may need to be maintained. To further understand these results, we tested if catalase would have beneficial effects in conditions of exacerbated levels of ROS. To achieve this, we optimised a feeding protocol for the delivery of the bipirydyl redox cycler, PQ, which promotes the formation of mitochondrial superoxide radicals [35, 36]. Flies were raised on standard food for 14-16 days, prior to exposure with PQ or vehicle dissolved in 2.5% sucrose/PBS solution. Flies were then fed PQ on alternating days over 8 days in total (final age = 22-25 days). Flies were returned to standard food on days without drug treatment to minimise the impact of a sucrose-only diet.

Flies fed with 5 mM PQ had a significant increase in the frequency of MT unbundling and axonal swellings in T1 neurons compared with aged-matched controls (Supplementary Fig. 1), confirming the detrimental effect of increased ROS on MTs and axonal health. In PQ-treated flies, overexpressing catalase fully rescued PQ-induced MT-associated phenotypes (Supplementary Fig. 1). Despite the protective effect observed from catalase overexpression on MTs when exposed to PQ, catalase overexpression did not significantly alter the frequency of axonal swellings (Supplementary Fig. 1). The lack of a protective effect of catalase overexpression on the swelling phenotype induced by PQ, could be explained by the greater frequency of axon swellings already observed in flies overexpressing catalase. Alternatively, distinct ROS species (hydrogen peroxide versus superoxide) may have differential effects and or requirements in axons.

**Figure 4.**
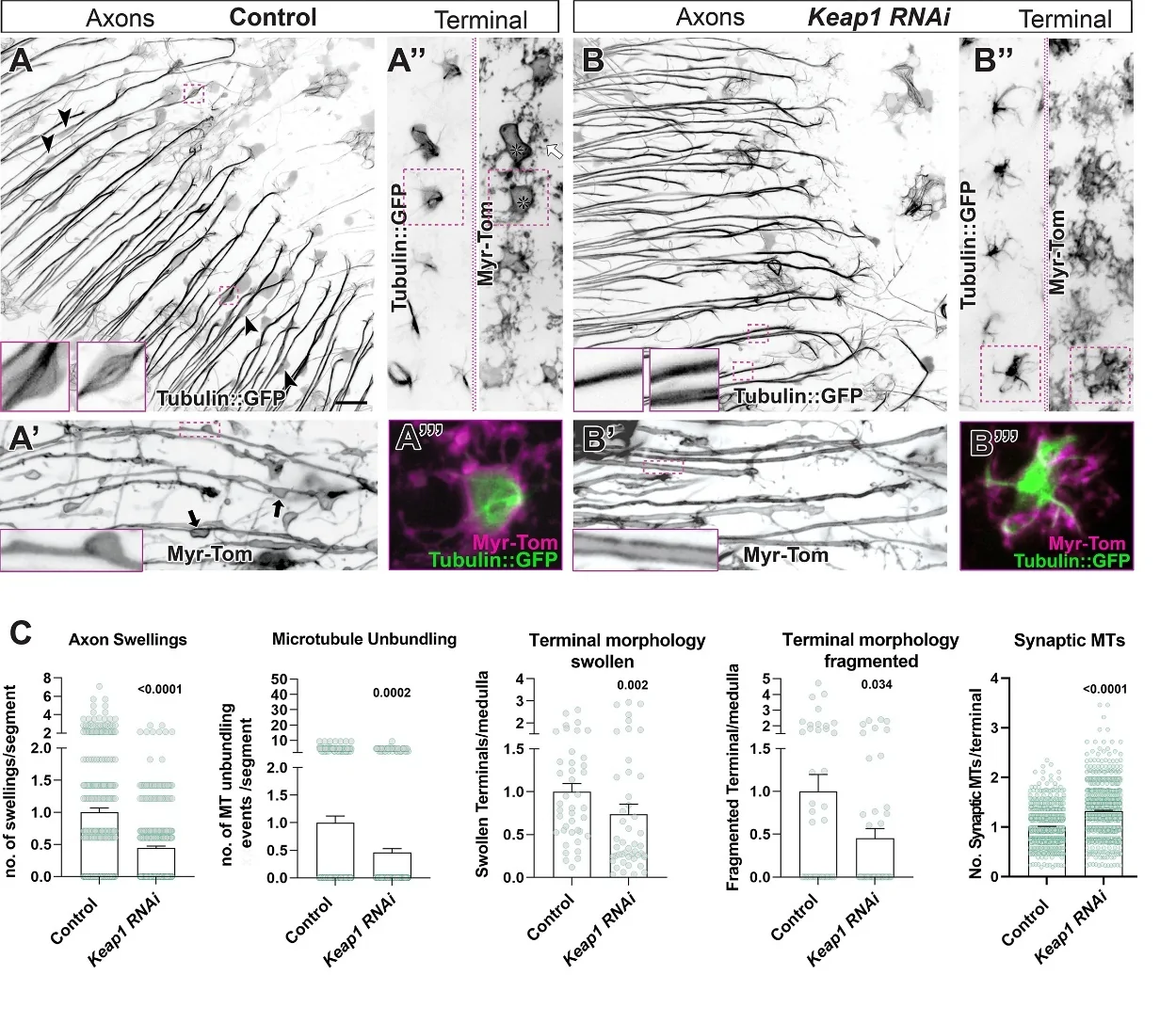
**Knocking down *Keap1* attenuates the onset of age-related microtubule alterations and morphological changes in axons and terminal**. (A-B’) Medulla regions of adult brains aged 30-40 days post eclosure, which show T1 axons labelled with GFP-tagged α-tubulin (tubulin::GFP) and the plasma membrane marker myristoylated-Tomato (myr-Tom). Aged neurons in the absence (A) or presence of *Keap1* knockdown (*Keap1 RNAi*; B) are compared. In axons (A-B’) *Keap1* knockdown supresses the appearance of microtubule unbundling and axonal swellings (black arrow heads for microtubule unbundling and black arrows for swellings in A and A’ compared with B and B’). *Keap1* knockdown also improves axon-terminal phenotypes (A’’-B’’’) as contains more synaptic microtubules and less swollen and broken terminal morphologies (asterisk for swollen and white arrows for broken synapses in A’’ compared to B’’ and insets with magnified images of a terminal in A’’’ and B’’’). (**C**) Quantifications of phenotypes described above; bars represent normalised mean ± SEM; data points are shown in green; p values are shown above each bar, as assessed by Mann-Whitney rank sum tests. For the assessment of axon swellings and microtubule unbundling, data were collated across two individual repeats, from a minimum of 300 axonal segments from 15 control and 20 *Keap1 RNAi* medullas. For the assessment of axon terminal phenotypes, data were generated from four individual repeats, from a minimum of 700 synapses from 41 control and 47 *Keap1 RNAi* medullas. Scale bars = 10 μm.

Our second approach aimed to enhance the cellular antioxidant defence system was to express *UAS-Keap1 RNAi to* increase NRF2 activity [37]. We found that *Keap1* knockdown in T1 neurons significantly reduced the frequency of MT unbundling and axonal swellings in aged flies compared with aged-matched controls (Fig. 4). In addition, *Keap1* knockdown halted the morphological deterioration of synaptic terminals; a significantly greater number of MTs observed at synaptic sites in Keap1 RNAi flies compared with age-matched controls (Fig. 4). Taken together, these findings indicate *Keap1* knockdown prevents age-related MT deterioration and is associated with a reduction in axonal and synaptic atrophy. Alternatively, upregulation of catalase alone provided some beneficial effects to MT integrity in conditions of elevated ROS; however, an overall detrimental effect on the health of axons was observed.

### Oxidative stress alters microtubule organisation and stability in primary neuronal cultures

Given the ROS-induced changes in MTs observed using the *in vivo* T1-cell aging model, we proposed that MT-based physiology may be a key target of oxidative stress. To determine the processes whereby elevations in ROS impact MT physiology, we used *Drosophila* primary neuronal cell cultures, established in previous work, as an *in vitro* neuronal model to study MTs [38].

**Figure 5.**
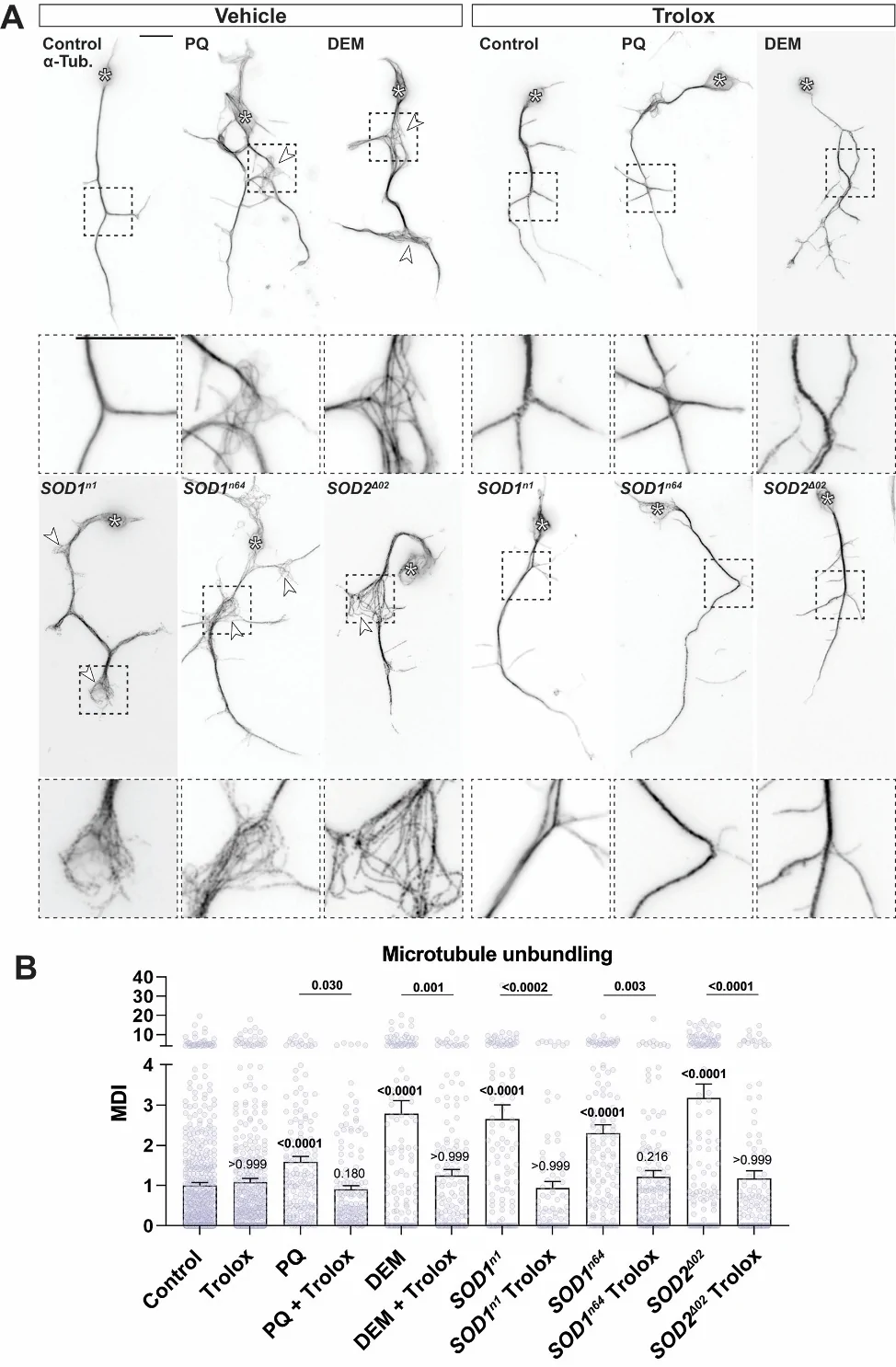
**Increased ROS promote microtubule disorganisation in primary neuronal cultures**. (**A**) Representative images of 6 DIV *Drosophila* primary neurons stained for tubulin. Neurons were cultured in different conditions: control treated with 100 μM PQ, control treated with 100 μM DEM, and neurons carrying either of the mutant alleles *SOD1^n1^*, or *SOD1^n64^* or *SOD2^Δ02^* in homozygous. Cells were treated with ethanol (vehicle) or 100 μM trolox. Asterisks indicate cell bodies and white arrow heads indicate regions of microtubule disorganisation, magnified in dashed boxes. (**B**) Quantitative analysis of the microtubule disorganisation index (MDI). Bars represent normalised mean ± SEM. P values are shown above each bar, as assessed by a Kruskal-Wallis one-way test. Data were generated from 14 experiments with 3 individual cultures per condition per repeat, with a minimum of 100 overall neurons evaluated per condition. Scale bars = 10 μm.

PQ and DEM, which depletes GSH levels in cells [35, 36], have been previously utilised in research on oxidative stress [39, 40]. Here, we cultured *Drosophila* embryo derived primary neurons, which were treated with either vehicle, 100 μM PQ or 100 μM DEM at 3 days in vitro (DIV) and fixed and imaged at 6 DIV. A MT disorganisation index (area with MT disorganisation/axon length) was employed as a robust readout indicative of alterations in MT organisation [29, 41]. In both PQ- and DEM-treated neurons, significant MT curling and unbundling within axons was observed in comparison with vehicle-treated neurons (Fig. 5). This ‘MT disorganisation’ phenotype shared strong similarity to MT abnormalities we observed in aging T1 neurons (Fig. 1). To confirm whether this physiological disruption to MTs was mediated by excess ROS formation, we also co-treated neurons with the vitamin E analogue, Trolox, and PQ or DEM; Trolox is a potent ROS scavenger that exerts antioxidant effects [42, 43]. Co-administration of Trolox fully prevented the development of PQ- and DEM-induced MT disorganisation, indicating that dysregulation of MT organisation was mediated by elevated levels of ROS (Fig. 5).

To validate these findings and discard confounding toxicity or off-target effects from PQ and DEM as a potential cause of these phenotypes, we tested whether neurons deficient in SOD activity displayed similar MT aberrations. Despite lethality in the adult, harvesting neurons from *Drosophila* embryos facilitated the culturing of cells with homozygous *SOD*-mutant genes. Two amorphic mutant SOD1 alleles (*SOD1^n64^* and *SOD1^n1^;* [21]) were tested. Neurons harbouring either mutation exhibited severe MT curling and unbundling (Fig. 5). Likewise, homozygous *SOD2^Δ02^* neurons had significantly increased MT disorganisation compared with control cells. Taken together, these data suggest that loss of SOD antioxidant activity within cytosol or mitochondria is sufficient to disrupt MT bundling *in vitro*. Further supporting the relationship between ROS levels and MT unbundling, we found that trolox supplementation, administered during plating (0 DIV) and refreshed at 3 DIV, significantly prevented the onset of this MT disorganisation in both SOD1- and SOD2-mutant neurons (Fig. 5).

Given the dramatic change in MT organisation in response to oxidative stress, we investigated whether ROS impacts MT stability using a previously reported nocodazole-sensitivity assay as a proxy readout of MT stability in primary *Drosophila* neurons [44, 45]. Nocodazole is a reversible agent that through its interaction with β-tubulin inhibits MT assembly [44]. Since MT polymerisation is inhibited in the presence of nocodazole, it is possible to assess whether existing MTs are labile or stable.

**Figure 6.**
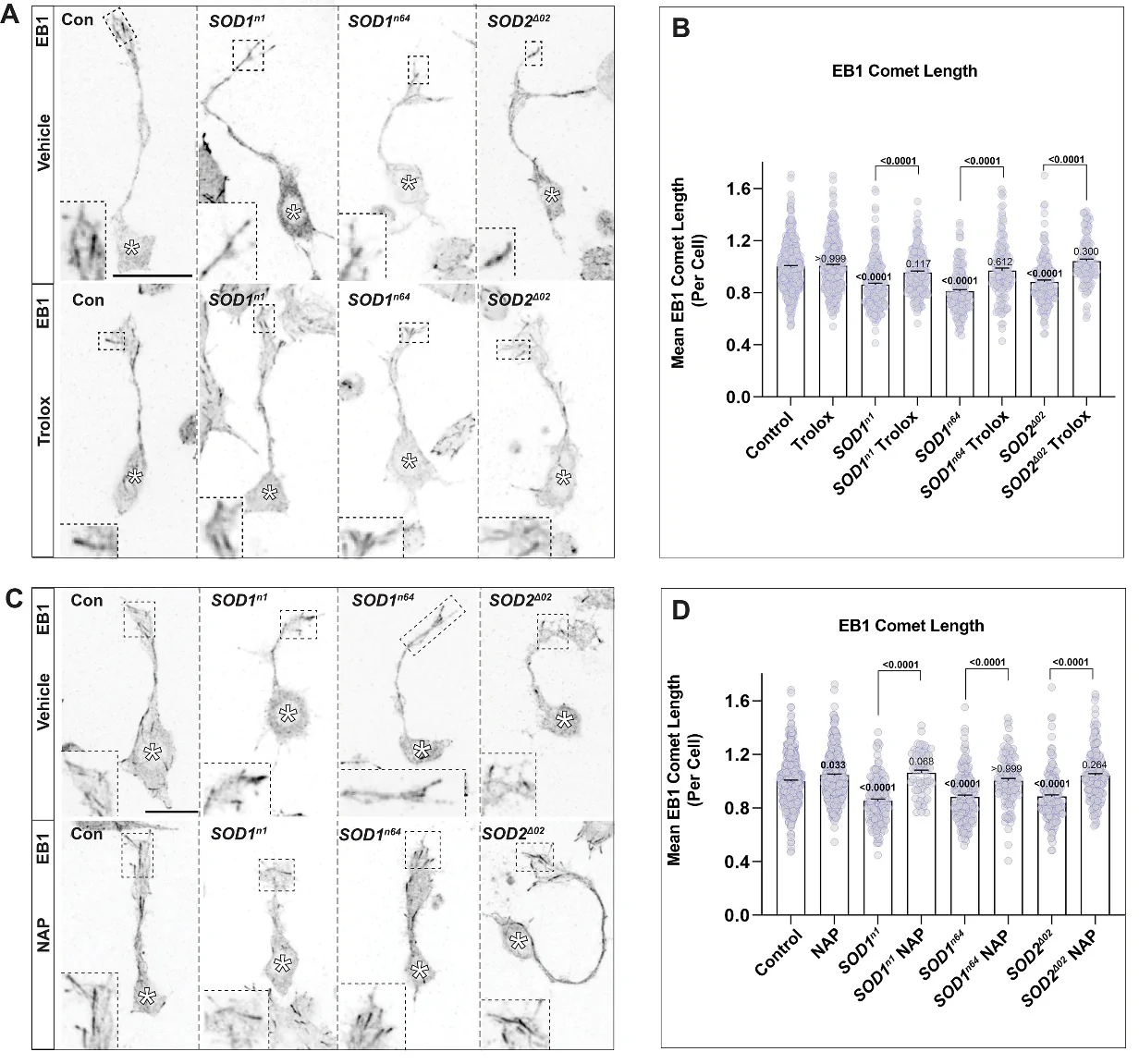
**Increased levels of ROS reduce EB1 comet length in primary neurons and this can be rescued by NAP**. (A and C) Representative images of 6 hours *in vitro Drosophila* primary neurons stained for EB1. Neurons of different genotypes: *w^1118^* (control) or carrying the *SOD1^n1^* or *SOD1^n64^* or *SOD2^Δ02^* mutant allele in homozygous. Cells were treated with ethanol or H_2_O (vehicle in A and C, respectively) or 100 μM Trolox (A) or 1 nM NAP (C) during plating (0 hours *in vitro*); Asterisks indicate cell bodies and dashed boxes show regions of EB1 comets, magnified in the respective inset. (B and D) Quantitative analysis of EB1 comet lengths per cell; bars represent mean ± SEM normalised to control; data points are shown in blue; P values are shown above each bar, as assessed by a Kruskal-Wallis one-way test. Data were generated from six independent repeats with 3 individual cultures per condition per repeat, with a minimum of 120 neurons (B) or 60 neurons (D) evaluated per condition. Scale bar = 10 μm

In primary neurons incubated with 100 μM nocodazole for 5-6 hours, the number of regions within axons where MTs were absent (as consequence of depolymerisation) were recorded and quantified per cell. These regions devoid of MTs often appear as ‘gaps’ or ‘breaks’ within MT bundles and were used to infer a decrease in MT stability to nocodazole. We found that MT gaps could be seen in control cells treated with nocodazole; however, significantly greater numbers of MT gaps were observed in SOD1- or SOD2-mutant neurons, suggesting that excessive ROS renders MTs less stable (Supplementary Fig. 2). Additionally, we found a small exacerbation in the number of MT gaps in neurons co-treated with nocodazole and DEM for 5-6 hours. In neurons pre-treated with DEM for 12-13 hours, before co-treatment with nocodazole and DEM for the 5-6-hour period, a substantial increase in the number of MT gaps was observed compared with DEM alone or nocodazole alone. This elevation in MT gaps was significantly enhanced in comparison with the shorter DEM incubation time (Supplementary Fig. 2 A and C).

Taken together, these data suggest that excessive ROS disrupts the organisation and decreases the stability of axonal MTs.

**Figure 7.**
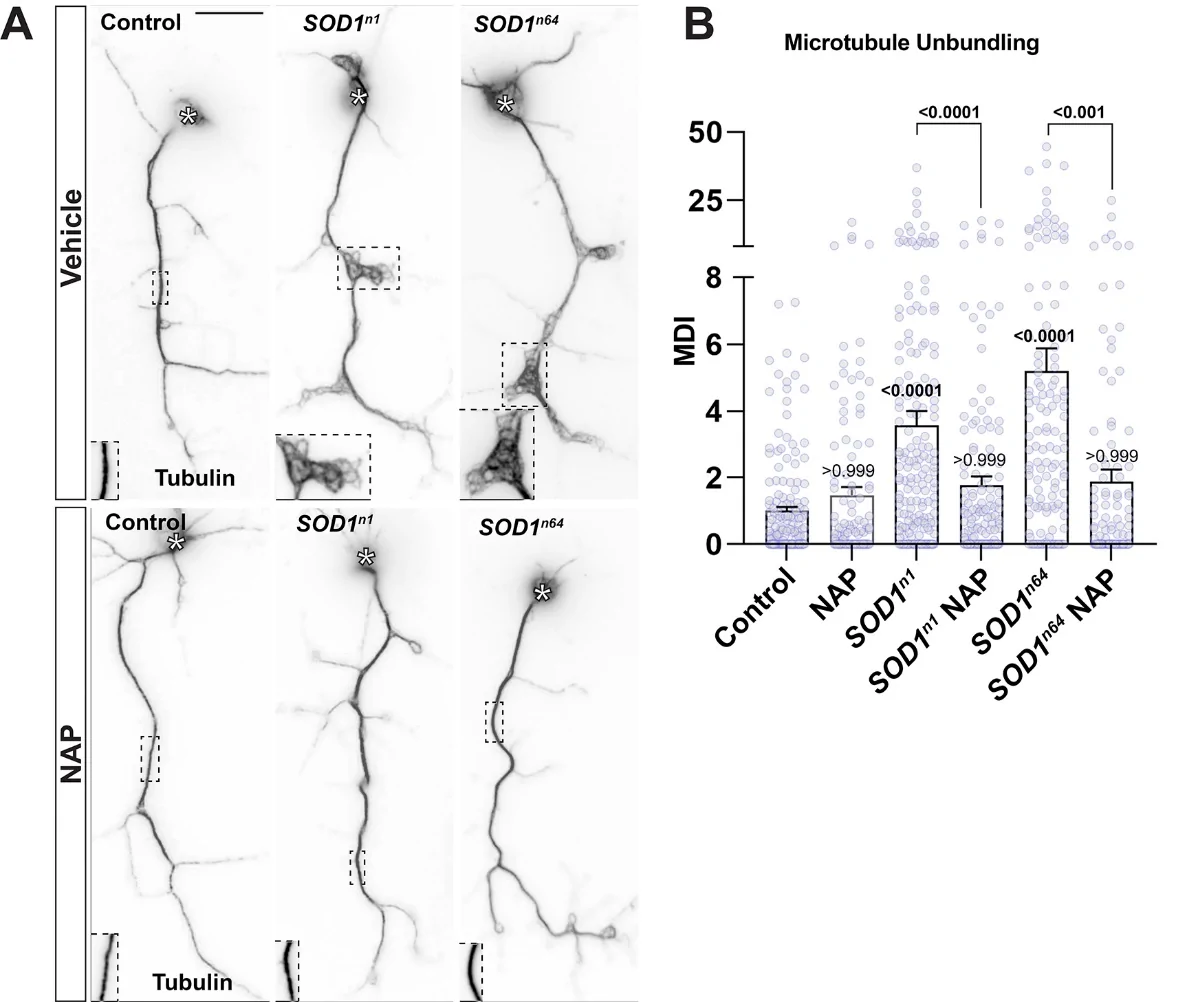
**NAP rescues ROS-induced microtubule disorganisation in primary neurons**. (**A**) Representative images of primary *Drosophila* neurons stained for tubulin. Neurons of different genotypes: *w^1118^* (control) or carrying the *SOD1^n1^*- or *SOD1^n64^*-mutant allele in homozygosis. Cells were treated with H_2_O or 1 nM NAP at 6 hours *in vitro* and refreshed at 3 DIV; asterisks indicate cells bodies, regions of microtubule disorganisation within dashed boxes are shown magnified in the respective inset. (**B**) Quantitative analysis of MDI per cell. Bars represent normalised mean ± SEM; data points are shown in blue; P values are shown above each bar, as assessed by a Kruskal-Wallis one-way test. Data were generated from four independent repeats with 3 individual cultures per condition per repeat and a minimum of 120 neurons per condition. Scale bar = 10 μm.

### MT alterations caused by oxidative stress are mediated through changes in EB1 in cultured neurons

At this stage, we had established a clear relationship between increasing levels of ROS and disrupted MT physiology; however, the mechanism(s) whereby ROS impacts MTs remains unknown.

MT polymerisation predominantly occurs at the MT plus end and is regulated by the family of MT plus end-binding proteins (EBs) [46]. The high affinity of EBs for GTP-bound tubulin results in their transient binding to plus ends of growing MTs; this activity leads to the formation of dynamic and characteristic ‘comet’-like foci. *In vitro* polymerisation assays have demonstrated that EB1 regulates MT dynamics and stability, and investigations in *Drosophila* neurons showed that a reduction in EB1 comet size correlates with an increase in MT disorganisation [29, 47, 48]. Given that EB1 is a key MT regulator in *Drosophila* neurons, we hypothesised that the effects of oxidative stress observed on MTs could be mediated through changes in EB1.

To investigate the impact of ROS on EB1 function, we first assessed the ability of EB1 to bind to MT plus ends. We visualised endogenous EB1 comets in primary neuronal cultures by immunohistochemistry and measured the mean comet length as a proxy measure of EB1 recruitment to MT plus ends. Control and *SOD1*- or *SOD2*-mutant neurons were compared at 6 hours *in vitro* because, at this timepoint, EB1 comets are prominent and easy to evaluate. We found that in neurons homozygous for *SOD1^n64^*, *SOD1^n1^* or *SOD2^Δ02^*, axonal EB1 comet length was significantly reduced compared with control neurons (Fig. 6). Furthermore, comet length was fully rescued when these mutants were supplemented with Trolox at plating (Fig. 6 A and B), suggesting that increasing ROS levels are inversely associated with EB1 comet size. We, therefore, proposed that ROS at higher-than-normal levels impair EB1 recruitment on MTs and hypothesised that MT defects induced by oxidative stress are driven by changes in EB1 physiology at the MT plus end.

To confirm our hypothesis, we considered that enhancing EB1 activity or its affinity for the MT plus end could rescue ROS-induced reductions in EB1 comet size, and, in turn, ROS-induced MT defects. To test this, we utilised the neuroprotective peptide NAPVSIPQ (NAP), derived from activity-dependent neuroprotective protein (ADNP), which exhibits an EB1-interacting SxIP motif [49, 50]. In differentiated neuroblastoma N1E-115 cells, it has been shown that NAP strongly enhances EB dynamics and comet length, through modulation of EBs function [51]. In *SOD1*- and *SOD2*-mutant neurons, we found that 1 nM NAP treatment fully rescued ROS-induced EB1 comet shortening compared with control neurons (Fig. 6 C and D). Remarkably, NAP supplementation also fully prevented ROS-induced MT disorganisation (Fig. 7). These data suggest that dysregulation of MTs by oxidative stress can be rescued by supporting EB1 binding at MT plus ends without a need to boost antioxidant capacity; therefore, changes in EB1 function may be responsible for ROS-induced MT unbundling. Supportive of these findings, we demonstrated that overexpression of EB1 (*elavGal4; UAS-Eb1::GFP*) in primary neurons could also prevent DEM-induced MT disorganisation (Supplementary Fig. 3 A and C). Conversely, we showed that the effects of DEM in MT disorganisation, was exacerbated in neurons homozygotes for *eb1^04524^*. *eb1^04524^* is an hypomorphic allele that maintains EB1 protein levels albeit reduced [25, 29, 52], the fact that DEM leads to greater microtubule disorganisation in this mutant background, suggests that reduced levels of EB1 increases sensitivity to ROS.

### Enhancing EB1 protects against oxidative stress *in vivo*

**Figure 8.**
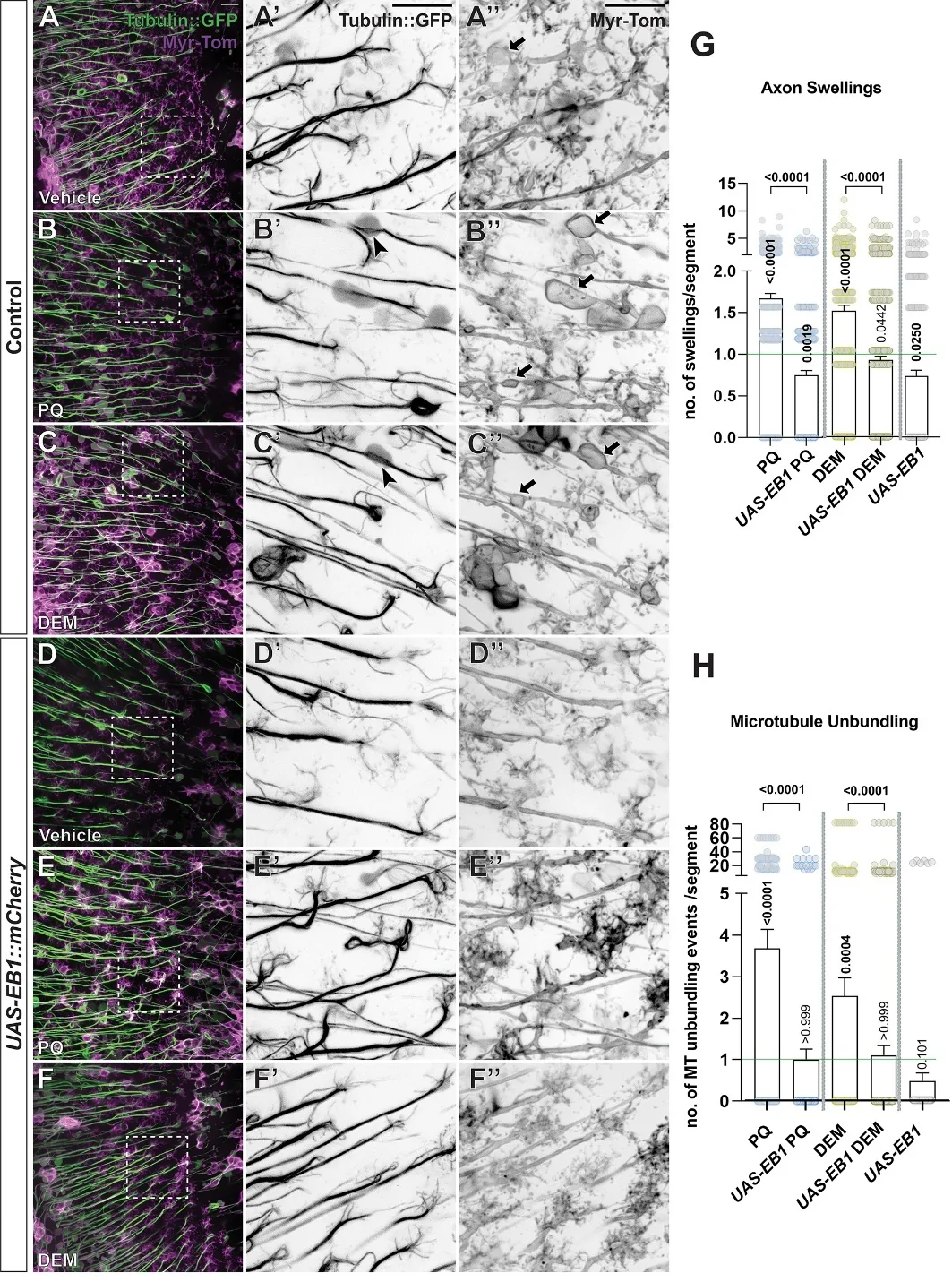
**Overexpression of EB1 prevents oxidative stress-induced microtubule unbundling and axonal swellings in the aged brain. (A–F”)** Medulla region of adult brains at 22–25 days post eclosure, depicting T1 axons labelled with GFP-tagged α-tubulin (tubulin::GFP) and the plasma membrane marker myristoylated-Tomato (myr-Tom). At 14–17 days post eclosure, flies were treated with vehicle (**A, D**) or 5 mM PQ (**B, E**) or 2.5 mM DEM (**C, F**) in 2.5% sucrose every alternate day. Magnified images of regions, outlined by dashed white boxes are shown for tubulin::GFP (**A’, B’, C’, D’, E’, F’**) and Myr-Tom (**A”, B”, C”, D”, E”, F”**) as inverted greyscale images. Aged neurons in the absence (**A–C”**) or presence (**D–F”**) of EB1::mCherry overexpression are compared. EB1 overexpression supresses oxidative stress induced phenotypes in aging neurons, comprising axon swellings (arrows) and MT unbundling (arrow heads); boxed areas shown as magnified images below. Black arrows indicate regions of axonal swellings and black arrow heads indicate regions of microtubule disorganisation. (**G–H**) Quantitative analyses of the frequency of axonal swellings and microtubule unbundling. Conditions with drug treatment have been normalised and compared to internal vehicle-treated controls (green line). Bars represent normalised mean ± SEM; data points are shown in coloured circles; P values are shown above each bar, as assessed by Kruskal-Wallis one-way tests. Data were generated from 4 repeats for experiments involving PQ and 5 repeats in experiments involving DEM. Number of medullas assessed per group were 35 in PQ (5 mM), 32 in DEM (2.5 mM), 24 in UAS-EB1::mCherry, 21 in UASEB1:: mCherry PQ and 38 in UAS-EB1::mCherry DEM. A minimum of 300 axonal segments were evaluated per condition. Scale bars = 10 μm.

**Figure 9.**
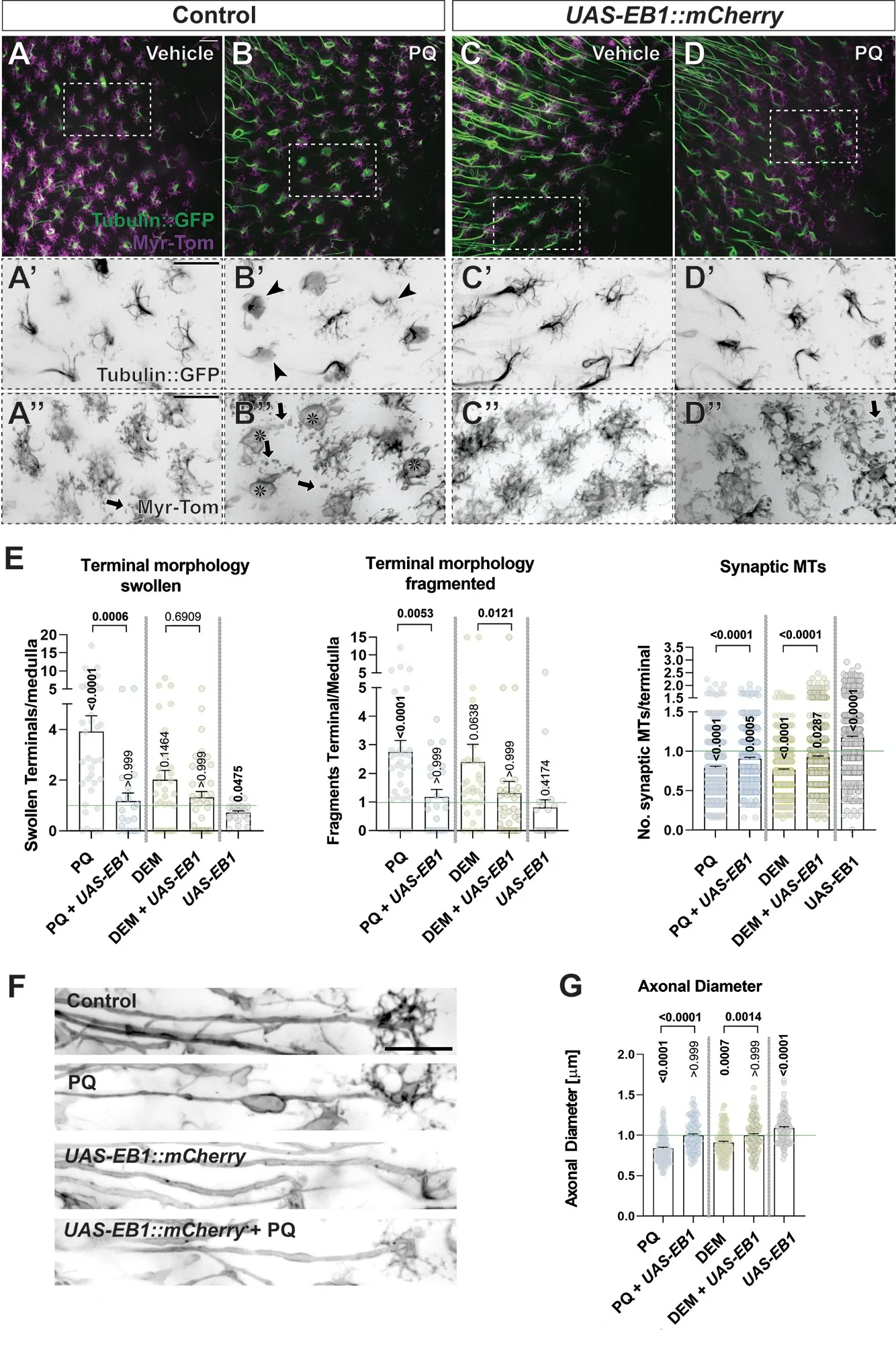
**Oxidative stress-induced synaptic terminal aberrations and axonal thinning is prevented by overexpression of EB1**. (**A-D**) Medulla region of adult brains at 22-25 days post eclosure, depicting T1 axons labelled with GFP-tagged α-tubulin (tubulin::GFP) and the plasma membrane marker myristoylated-Tomato (myr-Tom). At 14-17 days post eclosure, flies were treated with vehicle (A, C) or 5 mM PQ (B, D) in 2.5% sucrose every alternate day. Magnified images of regions, outlined by dashed white boxes are shown for tubulin::GFP (A’, B’, C’, D’) and Myr-Tom (A’’, B’’, C’’, D’’) as inverted greyscale images for easier visualisation. Aged neurons in the absence (A-B’’) or presence (C-D’’) of EB1::mCherry overexpression and with different oxidant treatments are compared. PQ induced aging-related phenotypes at synaptic terminals, including an increase in swollen (asterisks) and broken terminals (arrows), and a decrease in synaptic MTs (arrowheads), are supressed upon EB1 overexpression in T1 neurons. (**F**) Representative images of T1 axonal membranes labelled with myristoylated-Tomato and under same conditions as indicated above. Note PQ induces a decrease of the diameter of axons which can be counteracted by EB1::mCherry overexpression. (E and G) Quantitative analyses using the conditions above as well as DEM treatments, of the proportion of synaptic terminal swellings per medulla, fragmented synaptic terminals per medulla, mean number of synaptic microtubules per terminal and axonal diameter. Conditions with drug treatment have been normalised and compared to internal vehicle-treated controls (green line). Bars represent normalised mean ± SEM; data points are shown in coloured circles; P values are shown above each bar, as assessed by Kruskal-Wallis oneway tests. Data were generated from 4 individual repeats per condition, with a minimum of 20 medullas evaluated per condition. Scale bars = 10 μm.

Given that we had shown that enhancing EB1 protects against the effects of ROS in neurons *in vitro*, we investigated whether the same relationship could be observed *in vivo* using the T1 aging model. We utilised the protocol described above to feed PQ and DEM to flies and evaluate its effects in the T1 model. Both 2.5 mM DEM or 5 mM PQ treatment resulted in significant enhancement of aging phenotypes in T1 cells, including MT unbundling, axonal swellings, and swollen synaptic terminals (Fig. 8 and Fig. 9). In flies overexpressing EB1 in T1 neurons (*UAS-EB1::mCherry*) fed with 2.5 mM DEM or 5 mM PQ, we strikingly observed full suppression of PQ- and DEM-induced MT unbundling and axonal swellings (Fig. 8). In addition to this, we also observed widespread improvements in synaptic morphology and synaptic MTs (Fig. 9 A-F).

Previous work has shown that axon diameter is impacted by aging, which is also evident in the T1 aging model [12]. Consistent with PQ and DEM exacerbating aging hallmarks, we observed a significant decrease in mean axonal diameter compared with age-matched, vehicle-fed control flies (Fig. 9 F-G). Overexpression of EB1 prevented PQ-and DEM-induced reductions in axonal diameter (Fig. 9 F-G).

In summary, these data show that EB1 overexpression protects against oxidative stress-induced hallmarks of aging *in vivo*. Furthermore, we showed that axonal and synaptic deterioration induced by oxidative stress is caused by the dysregulation of the MT cytoskeleton, which involves changes in MT plus end physiology.

## DISCUSSION

Here, we aimed to investigate mechanisms whereby oxidative stress may impact neuronal physiology and aging. Our results showed that both pharmacological (PQ and DEM treatment) and genetic (*SOD1/2*-mutant strains; *SOD1* and *GSS1 and 2* knockdowns) induction of oxidative stress exacerbates hallmarks of aging in T1 neurons, which includes an increase in the frequency of axonal swellings, reduction in axonal diameter, and morphological transformation of axonal synaptic terminals with age. The discovery that MT deterioration observed in the aging brain [12], is exacerbated by oxidative stress, led to focus on the interplay between ROS and MT regulation. We further found that oxidative stress led to a prominent reduction in the binding of EB1 at MT plus ends in cultured neurons. By increasing EB1 activity, through the addition of NAP or EB1 overexpression, ROS-induced MT disorganisation could be fully prevented *in vitro*. A remarkable finding was that *in vivo* overexpression of EB1 using the aging T1 cell model could similarly fully prevent PQ-induced aging hallmarks in both axonal and synaptic compartments.

### Phenotypic hallmarks of neuronal aging are increased by oxidative stress

The onset of focal axonal swellings is considered a classical hallmark of aged nerve tissue and an early marker of Wallerian degeneration [53]. Prominent neuronal swellings, or blebbing, has been observed in aged brain tissue from mice, rat, primate, and humans [16, 54-56]. Notably, these swellings observed here in the *Drosophila* optic lobe resemble such cases found in other higher order animal models, suggesting convergent pathological consequences across species. High rates of axonal swellings are also observed in many neurodegenerative conditions, including AD, PD, ALS and multiple sclerosis, as well as in cases of traumatic brain injury (TBI) [57-61]; axon swellings are a typical sign of axonopathy [56, 62] hence their value as a readout for disease severity and, potentially, onset of age-related neurodegeneration. Our data show that the development of axonal swellings during aging is promoted by increasing ROS levels. This was supported through the depletion of SOD activity or downregulation of GSH production which accelerated T1 aging phenotypes. Additionally, by reducing Keap1 levels, thus enhancing NRF2 activity, the frequency of age-related axonal swellings were reduced.

Interestingly, PQ is a well-known herbicide and neurotoxicant that has been linked with the onset of sporadic PD [63-65]. The effects of PQ in our study, in the context of aging, may substantiate the impact of ROS in neurodegenerative disorders such as PD. In addition, studies show that NADPH oxidase activity is elevated as early as one-hour post-brain injury, suggesting a pathological role of ROS in the outcomes of TBI [66]. The extent of ROS-associated tissue damage and mitochondrial dysfunction in TBI patients correlates with more severe outcomes [67, 68]. Together, these findings could suggest that an increase in ROS following brain injury may trigger degenerative signalling pathways, predisposing individuals to AD and other neurodegenerative conditions [69-71].

### Oxidative stress, microtubule dysregulation and axonal atrophy

Another key finding from our study was that the onset of MT unbundling is induced by elevating ROS levels. We know from previous work that MT unbundling is an early aging hallmark observed in the T1 model [12]. In aged rhesus monkey brains, unbundled and disorganised MTs have been observed in axonal swellings, patently reminiscent of MT unbundling in T1 cells [16]. MT reduction has also been observed in biopsies of aged patients with and without AD, independent of Tau pathology [15]. Given these observations across organisms, we propose an aging cascade whereby oxidative stress triggers MT deterioration, driving axonal atrophy. Several lines of research demonstrate that MTs are vital for axonal integrity and our previous work has established that reducing the function of several MT-binding proteins leads to higher rates of axonal swellings during aging, and inversely, enhancing MTs decreases the prevalence of axonal swellings during aging [12].

The causative relationship between MT deterioration and axonal swellings is not unique to insect neurons, as this physiology has also been observed in vertebrates. For instance, nocodazole-induced MT disassembly in chick dorsal root ganglia [72] and MT decay, caused by loss of MAP1b in mouse Purkinje neurons [73], have been shown to promote the formation of axonal swellings. The ability of ROS to modify MT networks may be a common phenomenon found in higher organisms and in different cell types; for example, disorganised MTs have also been reported in human osteosarcoma cells and human umbilical cord vein endothelial cell cultures treated with H_2_O_2_ [74, 75]. Elevated ROS in myocytes has also been shown to promote MT depolymerisation [18].

Our work suggests that ROS affects MT regulation through a mechanism whereby excessive ROS hinders the ability of EB1 to bind to MT plus ends. We find that ROS-induced deficits in EB1 activity, and consequently in MT organisation, can be supressed by increasing EB1 levels or by supplementing neurons with the small neuroprotective peptide, NAP. NAP is derived from ADNP [76, 77], and previous work has shown that NAP rescues axonal transport and MT deficits in a *Drosophila* tauopathy model [78]. Furthermore, in a SOD1-G93A mouse model of ALS, NAP protected against disrupted axonal transport [79], and in PC12 cells, NAP had protective effects against oxidative stress [80]. Our study may explain how NAP protects against elevated ROS given that we observed a link between NAP treatment, EB1 binding to MT plus ends, and MT disorganisation. NAP (investigational drug: davunetide; also known as CP201) is under clinical investigation for neurodegenerative, MT-related tauopathies [81] as well as the autism-related ADNP syndrome [82].

The mechanisms of EB-dependent ROS-induced damage could extend to other cell types than neurons, as for instance, ROS in A549 cells and ventricular myocytes has been shown to reduce the accumulation of EB1 at MT plus ends [18, 83]. How ROS mediates its effects on MT-binding factors is open for discussion. Potential mechanisms could include direct thiol oxidation of EB1 [17, 84] or activation of redox-sensitive signalling pathways that are involved in the regulation of MTs, such as those that involve c-Jun N-terminal Kinase (JNK), apoptosis signal-regulating kinase (ASK1) [85], glycogen synthase kinase 3 (GSK3) [86] and p38 mitogen-activated protein kinases (p38 MAPK).

### The origin of ROS production in aging

One of the challenges in understanding the role of oxidative stress is aging is determining the source and type of ROS implicated in the aging process. For example, studies have shown that ROS elevations can enhance lifespan [87], which contradicts the general theory of oxidative stress as a causative factor in aging. A defining factor could be where oxidative stress is localised and the type of ROS produced. For example, our data using the T1 aging model suggest that *SOD2* heterozygote loss-of-function exhibits a greater acceleration of aging hallmarks compared with *SOD1* mutant heterozygotes; although, a mild increase in axonal swellings from the knockdown of *SOD1* was observed. This suggests that mitochondrial-localised ROS may have a greater impact on the aging process in the brain. Supportive of this, loss of Milton/Miro-mediated mitochondrial transport has been shown to enhance MT disorganisation in primary neuronal cultures [88]. In addition, we found that targeted knockdown of *Gss1 and 2* in T1 neurons also accelerated aging phenotypes showing that loss of glutathione metabolism could also be implicated in the aging process, and critically, that these effects are also cell autonomous. Interestingly, a study using *Drosophila* primary neuronal cultures showed that, in-addition to *SOD1* and *SOD2* loss-of-functions shown here, catalase loss-of-function and overexpression of NOX or Duox also promote MT unbundling in cultured neurons [88], suggesting that ROS-induced MT disorganisation may not necessarily be driven by subcellular specific origins of ROS.

Another interesting finding in our work was that GSH depletion by DEM treatment strongly induced MT unbundling and decreased MT stability. Previous research has shown that the depletion of mitochondrial GSH pools in cerebellar granule neurons (CGNs) stimulates ROS production and apoptotic pathways [89]. Moreover, the authors found that CGNs (and astrocytes) were particularly sensitive to GSH depletion in mitochondrial pools, as less of an impact was observed when depleting only cytosolic GSH, suggesting that mitochondrial GSH content may be fundamental for redox homeostasis in relation to apoptotic pathways [90, 91].

In support of the importance of GSH in healthy aging, we found that DEM exacerbated axonal swellings and other aging hallmarks in the T1 model [92, 93]. Other studies have shown that GSH depletion and elevated ROS are suspected to be an early preclinical marker of neuronal death in the substantia nigra during PD onset [93, 94], and altered GSH metabolism is apparent in hippocampal and cerebellar tissue from patients with AD [95].

In summary, the findings described here support a role of oxidative stress as a key driver of neuronal aging and suggest a novel role of ROS in sensitising the MT plus end. Manipulating MT-associated factors such as EB1 could be a novel and valuable therapeutic strategy to prevent the neurological decline observed with age and may also be a relevant therapeutic target in disease conditions with intrinsic oxidative stress.

## Supplementary Materials

The Supplementary data can be found online at: www.aginganddisease.org/EN/10.14336/AD.2024.0839.
