# Supplementary material for: Oxidative Stress Promotes Axonal Atrophy through Alterations in Microtubules and EB1 Function
Source: Aging Dis. 2024 Dec 3;16(6):3706–25. doi: 10.14336/AD.2024.0839 (PMC12539546; doi:10.14336/AD.2024.0839)
Supplement: Supplementary file 1 — The Supplementary data can be found online at: www.aginganddisease.org/EN/10.14336/AD.2024.0839. [file AD-16-6-3706-s.pdf]

SUPPLEMENTARY DATA

Oxidative Stress Promotes Axonal Atrophy through Alterations in Microtubules and EB1 Function

Samuel Shields, Emilia Gregory, Oliver Wilkes, Illana Gozes, Natalia Sanchez-Soriano

© 2024. Shields S. et al. Published online at http://www.aginganddisease.org/EN/10.14336/AD.2024.0839

SUPPLEMENTARY DATA

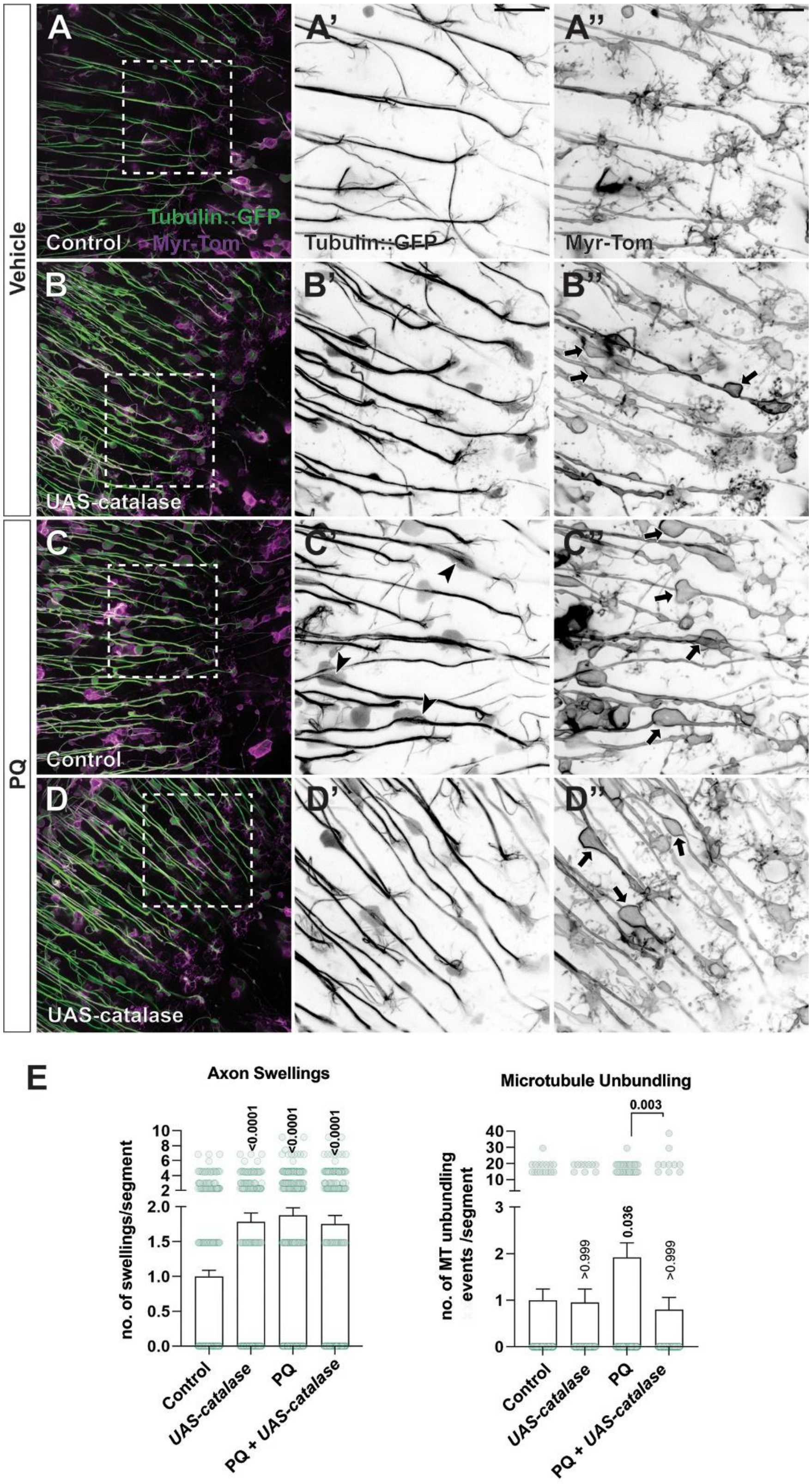

Supplementary Figure 1. Catalase overexpression rescues PQ-induced microtubule disorganisation. (A–D'') Medulla region of adult brains at 22–25 days post eclosure, depicting T1 axons labelled with GFP-tagged α-tubulin (tubulin::GFP) and the plasma membrane marker myristoylated-Tomato (myr-Tom). Flies without or with UAS-catalase expression in T1 neurons were treated at 14–17 days post eclosure with H_2O (A–B'') or 5 mM PQ (C–D'') in 2.5% sucrose every alternate day. Magnified images of regions, outlined by dashed white boxes are shown for tubulin::GFP (A', B', C' and D') and Myr-Tom (A'', B'', C'' and D'') as inverted greyscale images for easier visualisation. PQ induces microtubule disorganisation in axons which is prevented by catalase overexpression (black arrow heads in C' compared to D'). PQ also induces axonal swellings which cannot be rescued by catalase overexpression (black arrow in C'' and in D''). (E) Quantitative analyses of the phenotypes above. Bars represent normalised mean ± SEM; data points are shown in green; P values are shown above each bar, as assessed by Kruskal-Wallis one-way tests. Two individual repeats were performed with a total of 18 control, 12 UAS-catalase, 22 5 mM PQ and 17 UAS-catalase + PQ medullas assessed. A minimum of 180 axonal segments were evaluated. Scale bars = 10 µm.

© 2024. Shields S. et al. Published online at http://www.aginganddisease.org/EN/10.14336/AD.2024.0839

SUPPLEMENTARY DATA

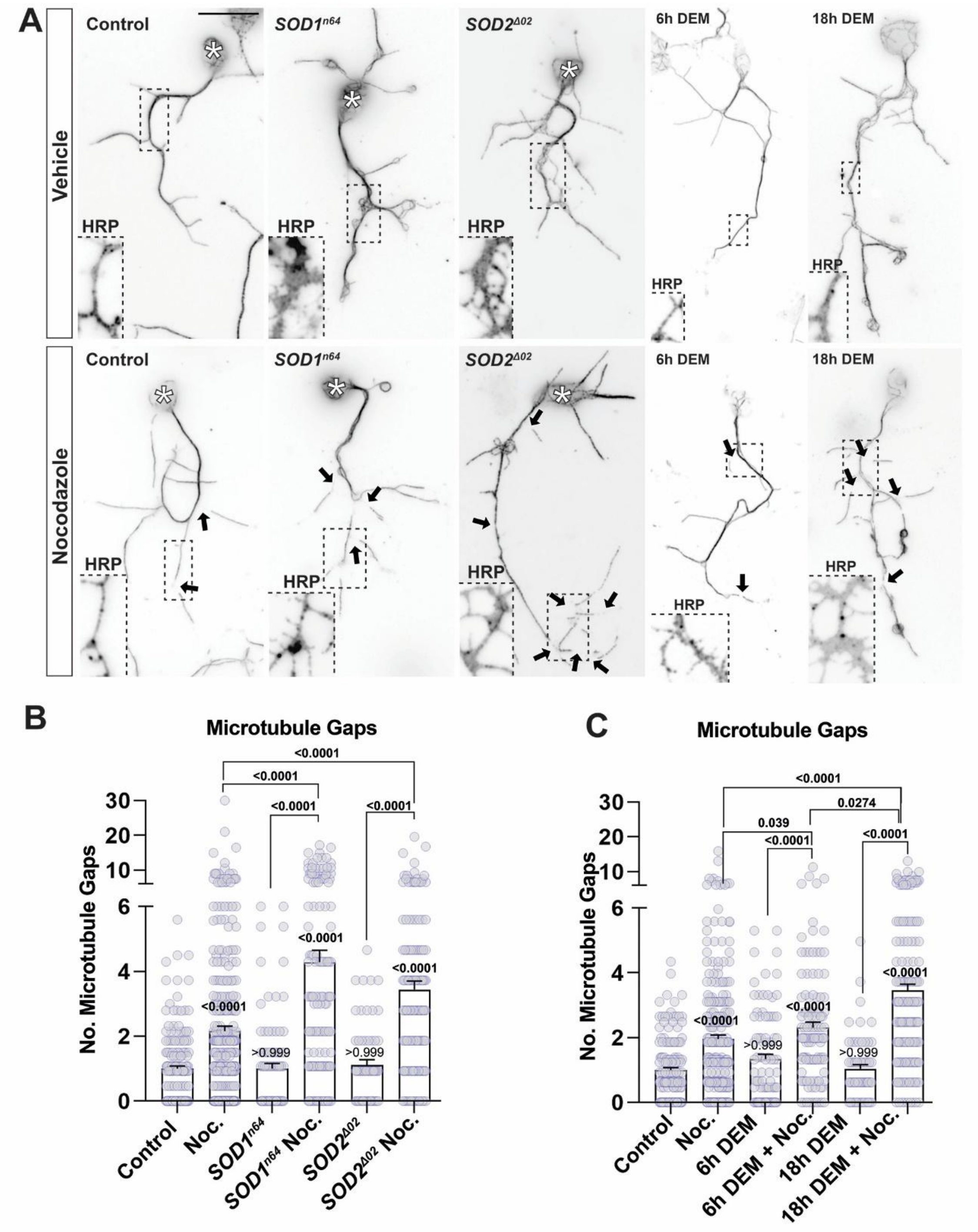

Supplementary Figure 2. Conditions of elevated ROS destabilises microtubules in primary neuronal cell cultures. (A) Representative images of 6DIV primary *Drosophila* neurons stained for tubulin and HRP to label microtubules and the cell membrane (only shown in the insets). Neurons of different genotypes: w^{1118} (control) or carrying the *SOD1^{n64}*- or *SOD2$^{\Delta02}$*-mutant allele in homozygous or treated with 100 µM DEM or ethanol at 6 HIV. Cells were treated with 100 µM nocodazole (Noc.) or DMSO at 18 HIV. Black arrows indicate sites of microtubule gaps, induced by nocodazole treatment, indicative of sensitive or unstable microtubules. Dashed boxes show regions of the axon that correspond to each image inset, which show continuous cell membrane using HRP staining. Asterisks label the cell bodies. (B) Quantification of the number of gaps in microtubules per cell; bars represent normalised mean ± SEM; data points are shown in blue; P values are shown above each bar, as assessed by Kruskal-Wallis one-way tests. Data were generated from five independent repeats. A minimum of 60 neuros per condition were evaluated. Scale bars = 10 µm.

© 2024. Shields S. et al. Published online at http://www.aginganddisease.org/EN/10.14336/AD.2024.0839

SUPPLEMENTARY DATA

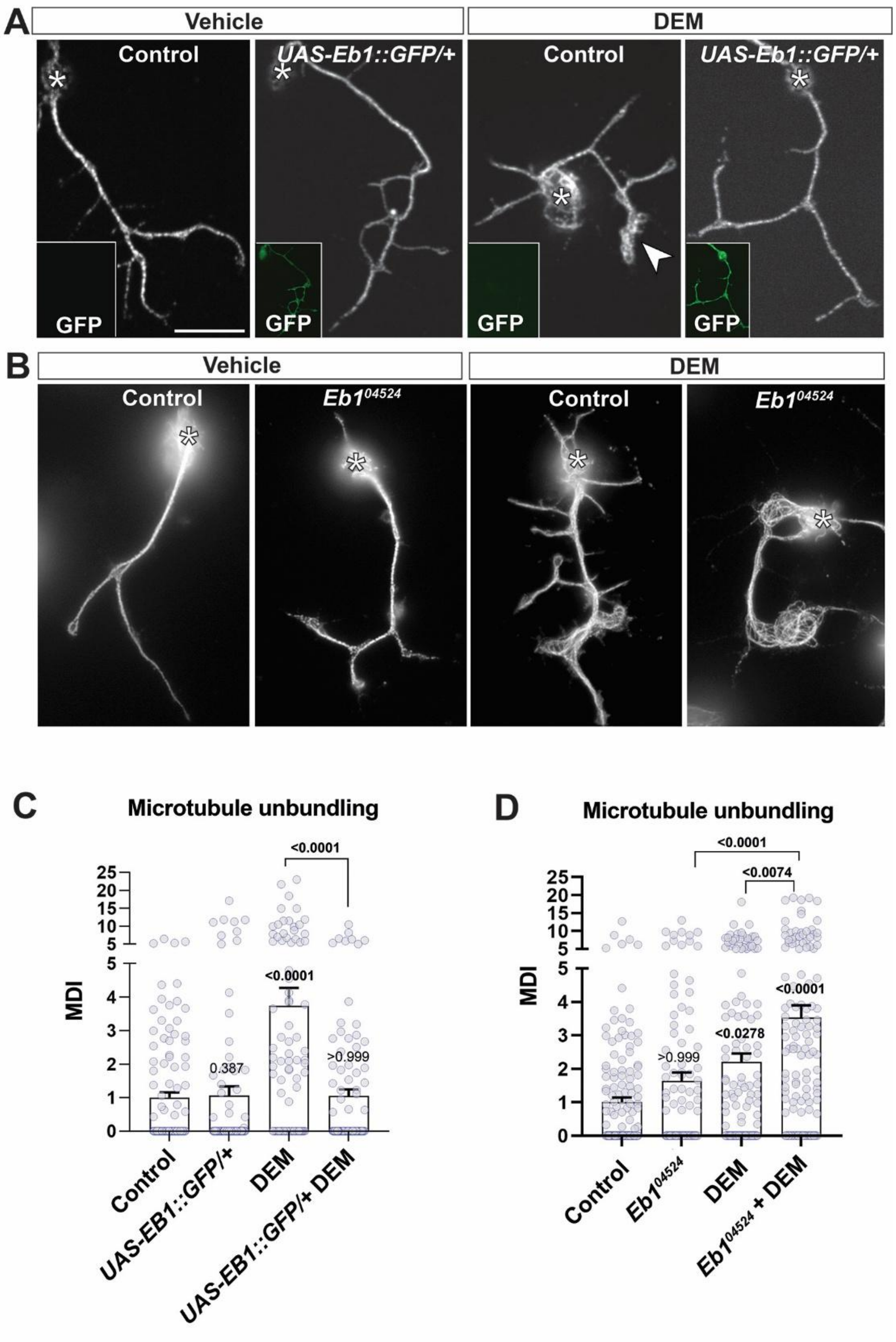

Supplementary Figure 3. Overexpression of EB1 prevents DEM-induced microtubule disorganisation. (A) Representative images of 6DIV primary *Drosophila* neurons stained for tubulin. Neurons of different genotypes: w^{1118} (control), overexpressing EB1::GFP or carrying the $EB1^{04524}$ mutation. Image insets in A shows whole-cell GFP signal (488 nm excitation). Cells were treated with ethanol or 100 µM DEM at 3 DIV. Asterisks indicate cells bodies and arrow heads indicate regions of microtubule disorganisation. (C and D) Quantitative analysis of MDI per cell; bars represent normalized mean ± SEM; data points are shown in blue; P values are shown above each bar, as assessed by Kruskal-Wallis one-way tests. Data were generated from 2 independent repeats with 3 individual culture per repeat. A minimum of 80 neuros per condition were quantified. Scale bar = 10 µm.

© 2024. Shields S. et al. Published online at http://www.aginganddisease.org/EN/10.14336/AD.2024.0839
